# The MluI Cell Cycle Box (MCB) Motifs, but Not Damage-Responsive Elements (DREs), Are Responsible for the Transcriptional Induction of the *rhp51* ^+^ Gene in Response to DNA Replication Stress

**DOI:** 10.1371/journal.pone.0111936

**Published:** 2014-11-05

**Authors:** Wugangerile Sartagul, Xin Zhou, Yuki Yamada, Ning Ma, Katsunori Tanaka, Tomoyuki Furuyashiki, Yan Ma

**Affiliations:** 1 Department of Oncology, First Affiliated Hospital of Liaoning Medical University, Jinzhou, China; 2 Division of Pharmacology, Kobe University Graduate School of Medicine, Kobe, Japan; 3 Department of Bioscience, School of Science and Technology, Kwansei Gakuin University, Sanda, Japan; University College London, United Kingdom

## Abstract

DNA replication stress induces the transcriptional activation of *rhp51* ^+^, a fission yeast *recA* homolog required for repair of DNA double strand breaks. However, the mechanism by which DNA replication stress activates *rhp51* ^+^ transcription is not understood. The promoter region of *rhp51* ^+^ contains two damage-responsive elements (DREs) and two MluI cell cycle box (MCB) motifs. Using luciferase reporter assays, we examined the role of these elements in *rhp51* ^+^ transcription. The full-length *rhp51* ^+^ promoter and a promoter fragment containing MCB motifs only, but not a fragment containing DREs, mediated transcriptional activation upon DNA replication stress. Removal of the MCB motifs from the *rhp51* ^+^ promoter abolished the induction of *rhp51* ^+^ transcription by DNA replication stress. Consistent with a role for MCB motifs in *rhp51* ^+^ transcription activation, deletion of the MBF (MCB-binding factor) co-repressors Nrm1 and Yox1 precluded *rhp51* ^+^ transcriptional induction in response to DNA replication stress. Using cells deficient in checkpoint signaling molecules, we found that the Rad3-Cds1/Chk1 pathway partially mediated *rhp51* ^+^ transcription in response to DNA replication stress, suggesting the involvement of unidentified checkpoint signaling pathways. Because MBF is critical for G1/S transcription, we examined how the cell cycle affected *rhp51* ^+^ transcription. The transcription of *rhp51* ^+^ and *cdc18* ^+^, an MBF-dependent G1/S gene, peaked simultaneously in synchronized *cdc25-22* cells. Furthermore, DNA replication stress maintained transcription of *rhp51* ^+^ similarly to *cdc18* ^+^. Collectively, these results suggest that MBF and its regulators mediate *rhp51* ^+^ transcription in response to DNA replication stress, and underlie *rhp51* ^+^ transcription at the G1/S transition.

## Introduction

Genomic stability is crucial for cell proliferation and survival, and its loss precipitates tumorigenesis in multicellular organisms. The integrity of the genome can be compromised by various environmental agents, such as UV irradiation and reactive chemicals, or by perturbed DNA replication during S phase. Depending on the type of perturbation, distinct DNA structure checkpoints are activated to maintain genomic integrity. For example, DNA damage outside S phase can delay entry to S phase and promote DNA repair during interphase or can induce programmed cell death to avoid passing mutations to daughter cells in multicellular organisms. On the other hand, stalled DNA replication forks resulting from substrate or energy deprivation during S phase cause DNA replication stress, which delays mitotic entry and inhibits the initiation of DNA replication from late replication origins. Genetic studies across species have identified intracellular signaling pathways that regulate DNA structure checkpoints. DNA damage outside S phase and DNA replication stress activate distinct, but partially overlapping, pathways. In fission yeast, Rad3 kinase primarily activates Chk1 and Cds1 upon DNA damage outside S phase and upon DNA replication stress, respectively. In turn, Chk1 and Cds1 regulate downstream effectors in distinct manners [1], [2]. Intracellular signaling pathways for DNA structure checkpoints appear to be conserved across species. In mammalian cells, ATM kinase activates the downstream kinases CHK1 and CHK2, which correspond to Cds1 and Chk1, respectively, in fission yeast [3].

Among its various actions, DNA replication stress regulates the transcription of multiple genes, perhaps to prevent genomic instability. Many of these genes, such as *cdc18* ^+^ and *cdc22* ^+^, are critical for and are induced upon the transition from G1 to S phase. Thus G1/S transcription and DNA replication stress maintain the transcription of these genes even after entry into S phase. In fission yeast, G1/S transcription depends on the transcription factor complex MluI cell cycle box (MCB)-binding factor (MBF), which is analogous to members of the E2F transcription factor family in metazoans [4]–[6]. MBF regulates the transcription of its target genes by binding to specific DNA motifs called MCB motifs in their promoters. MBF binds to MCB motifs throughout the cell cycle [7], but it is maintained in an inactive state by two co-repressors, Nrm1 and Yox1 [8]–[12]. During the G1/S transition, these co-repressors dissociate from MBF when phosphorylation by Cds1, thereby de-repressing MBF-mediated G1/S transcription [8]–[12]. DNA replication stress de-represses MBF-mediated transcription through a similar mechanism via the Rad3-Cds1 pathway [8]–[12]. Interestingly, DNA damage inactivates MBF-mediated transcription through the Chk1-mediated phosphorylation of Cdc10, an MBF component, highlighting the differential effects of DNA replication stress and DNA damage [13].

DNA replication stress induces the transcription of genes associated with functions other than cell cycle control. In fission yeast, *rhp51* ^+^, a homolog of *recA* in bacteria and *RAD51* in budding yeast, is one such gene. Rhp51 plays a critical role in the repair of DNA double strand breaks. The promoter of *rhp51* ^+^ contains at least two damage-responsive elements (DREs) and two MCB motifs. Shim *et al*. demonstrated that DNA damage induces *rhp51* ^+^ transcription through the binding of the zinc finger protein Rdp1 to DREs in the *rhp51* ^+^ promoter [14]. Caetano *et al. l*. demonstrated that DNA replication stress induces *rhp51* ^+^ transcription via phosphorylation of the MBF repressor Yox1p [8]. However, it is not known how DNA replication stress induces *rhp51* ^+^ transcription, or whether MBF binding to MCB motifs in the *rhp51* ^+^ promoter is critical for DNA replication stress-induced *rhp51* ^+^ transcription. Using a luciferase reporter assay, we found that MCB motifs, but not DREs, in the *rhp51* ^+^ promoter were responsible for the transcriptional induction of *rhp51* ^+^ in response to DNA replication stress. Furthermore, studies of cells deficient in checkpoint signaling molecules suggested that unidentified checkpoint pathways, other than the Rad3-Cds1/Chk1 pathway, contributed to the transcriptional regulation of *rhp51* ^+^.

## Materials and Methods

### Strains, media, and genetic and molecular biology methods

The *Schizosaccharomyces pombe* strains used in this study are listed in Table 1. The media, denotation and genetic methods have been described previously [15], [16]. Gene disruptions are indicated by the gene symbol preceded by Δ (for example, Δ*rhp51*). Proteins are denoted by Roman letters with only the first letter capitalized (for example, Rhp51).

**Table 1:** *Schizosaccharomyces pombe* haploid strains used in this study.

Strain	Genotype	Reference
HM123	*h^-^ leu1-32*	Our stock
KP6468	*h* ^+^ *leu1-32 nrm1*::*KanMX*_4_	This study
KP6469	*h^-^ leu1-32 yox1*::*KanMX*_4_	This study
KP3348	*h^-^ leu1-32 ura4-D18 cds1*::*ura4^+^*	[27]
KP3349	*h^-^ leu1-32 ura4-D18 chk1*::*ura4^+^*	[27]
KP6467	*h^-^ leu1-32 ura4-D18 chk1*::*ura4^+^ cds1*::*KanMX*_4_	This study
KP4892	*h^-^ leu1-32 ura4-D18 rad3*::*ura4^+^*	[27]
KP204	*h^+^ leu1 cdc25-22*	[28]
KP456	*h^-^ leu1-32 ura4-D18*	Our stock
KP6609	*h* ^-^ *leu1-32 ura4-D18 cds1-5Flag-kanMX_6_*	This study

### Construction of reporter plasmids

**Figure 1:**
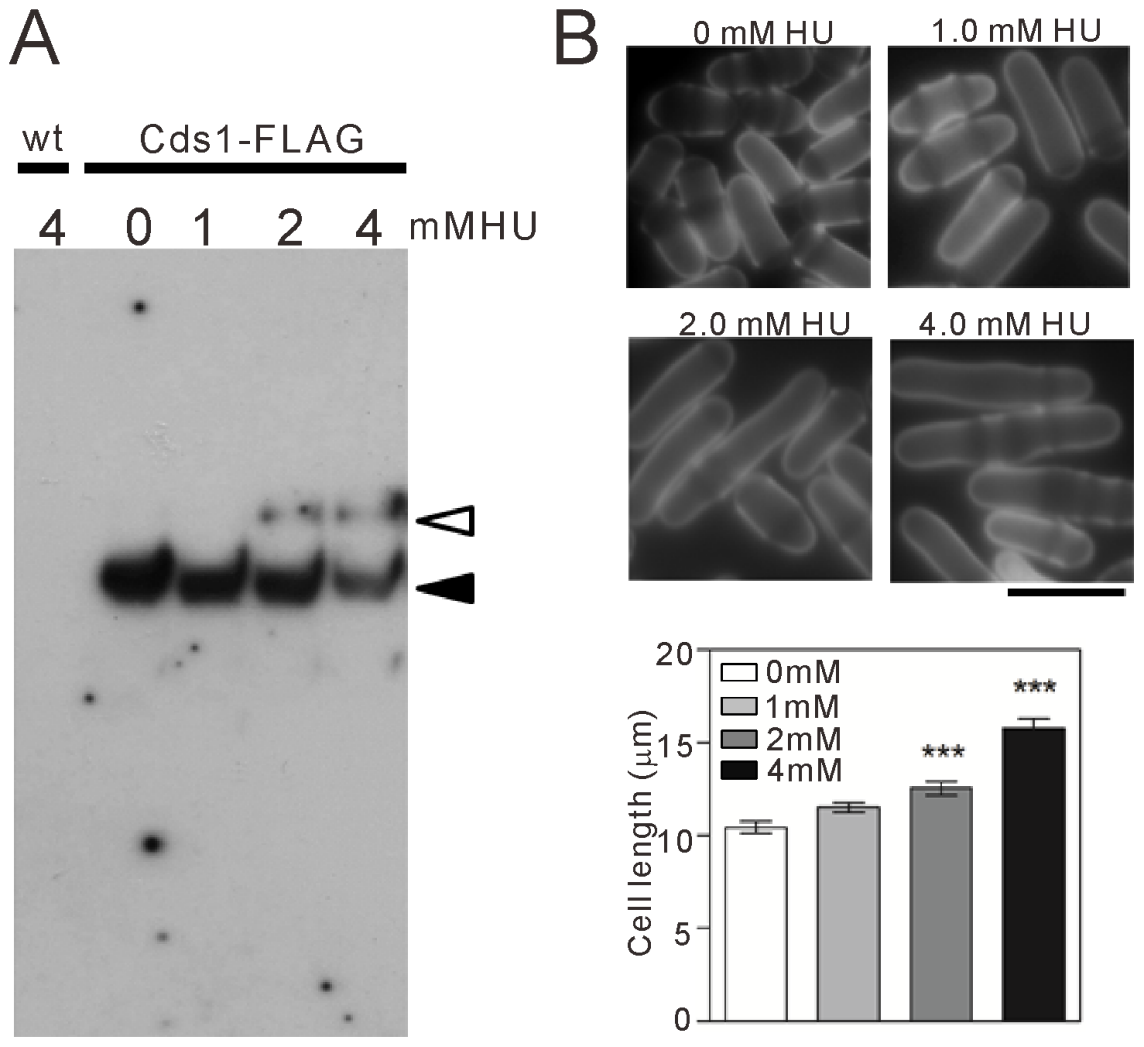
Induction of DNA replication stress upon HU treatment. *A*. Mobility shift detection of phosphorylated Cds1 upon HU treatment. Wild-type cells (“wt”; KP456) and cells expressing Flag-tagged Cds1 (“Cds1-Flag”; KP6609) were cultured to mid-log phase at 27°C in EMM supplemented with 225 mg/L leucine and uracil. The cells were divided into four equal volumes and incubated with HU at a final concentration of 1 mM, 2 mM, or 4 mM for 4 h. Cell lysates were subjected to Phos-tag SDS-PAGE, and Flag-tagged Cds1 was detected by immunoblotting with an anti-FLAG antibody. Because the migration of phosphorylated proteins is slower than that of non-phosphorylated proteins, the black and white arrows likely indicate the non-phosphorylated and phosphorylated forms of Cds1, respectively. Note that these signals were not detected in lysates from wild-type cells. *B.* Effect of HU treatment on cell length. Wild-type cells were cultured to mid-log phase at 27°C in EMM. The cells were divided into four equal volumes and incubated with HU at a final concentration of 1 mM, 2 mM, or 4 mM for 8 h. The cells were collected, stained with Calcofluor White, and observed under a fluorescence microscope. Scale bar, 10 µm. The cell lengths at each respective HU concentration were averaged, and the results are shown in the graph. *n* = 31 for each group. ***P<0.001 compared with the vehicle condition using one-way ANOVA followed by Tukey's test (*F*_(3,120)_  = 37.49, *P*<0.0001).

**Figure 2:**
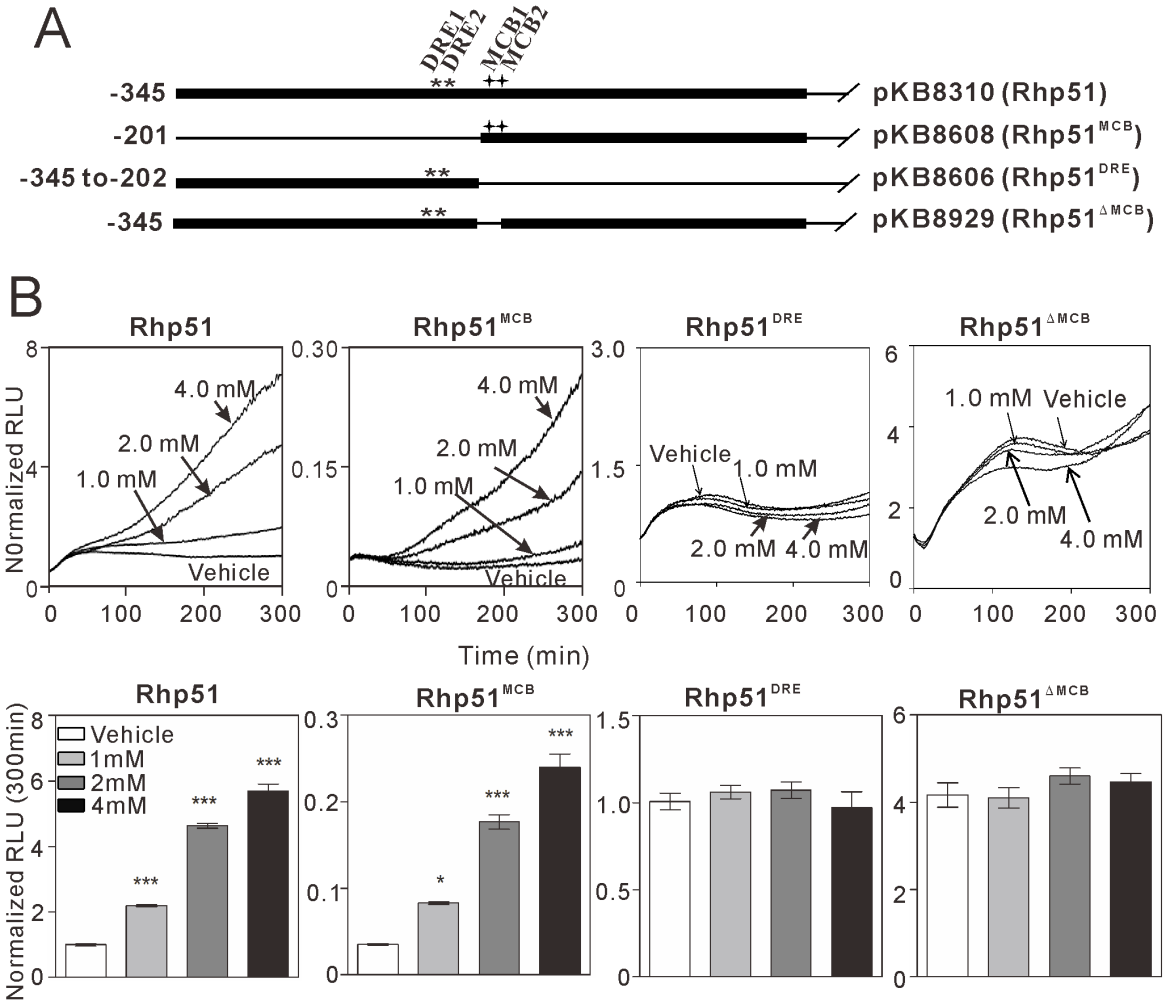
Real-time monitoring of *rhp51* ^+^ gene transcription in wild-type cells treated with HU. *A*. A schematic diagram of luciferase reporter vectors containing the full-length *rhp51* ^+^ promoter or *rhp51* ^+^ promoter deletion mutants. Two DRE decamers are located between bp −234 to −225 (DRE1) and bp −213 to −204 (DRE2) relative to the translation initiation site of the *rhp51* ^+^ promoter. Two MCB motifs are located between bp −192 to −187 (MCB1) and bp −183 to −178 (MCB2) in the *rhp51* ^+^ promoter. The following regions of the *rhp51* ^+^ promoter were inserted upstream of the open reading frame of luciferase: the full-length promoter ranging from bp −345 to −14 (pKB8310, designated Rhp51), a fragment from bp −201 to −14 containing two MCB motifs (pKB8608, designated Rhp51^MCB^), a fragment from bp −345 to −202 containing two DREs (pKB8606, designated Rhp51^DRE^), and the full-length promoter from which the two MCB motifs at bp −192 to −178 were deleted (pKB8929, designated Rhp51^ΔMCB^). *B*. Effect of HU on promoter activation. Wild-type cells transformed with the full-length *rhp51* ^+^ (Rhp51), Rhp51^MCB^, Rhp51^DRE^, or Rhp51^ΔMCB^ reporter were incubated with luciferin and then treated with HU (1 mM to 4 mM) for real-time monitoring of luciferase activity. Relative light units (RLU) were normalized to the values from wild-type cells harboring the full-length *rhp51* ^+^ reporter plasmid at 300 min without HU treatment. Representative traces of real-time monitoring are shown in the upper graphs. The lower graphs show the normalized RLU averaged across independent samples at 300 min in cells harboring the indicated reporter plasmids. *n* = 4 for each group. *P<0.05 and ***P<0.001 compared with the vehicle condition for the respective reporter using one-way ANOVA followed by Tukey's test.

A 332-bp DNA fragment in the 5′ flanking region of the *rhp51* ^+^ gene was amplified using the following PCR primers: sense primer 2737, 5′-AAA ACT GCA GGA CCA GTG CTG TTC TCT TGT TG -3′ and antisense primer 2738, 5′-CCG CTC GAG GCA CGA AAT TAT CAC TAT TCT GG-3′. The amplified products containing the 332-bp *rhp51* ^+^ promoter (−345 to −14, Figure 1A) were subcloned into a pGL3(R2.2)-basic multicopy vector (Promega) which contains a destabilized luciferase reporter gene, as described previously [17]. The resulting plasmid was registered as pKB8310 and used as the full-length *rhp51* ^+^ reporter vector. The truncated *rhp51* ^+^ promoter vectors were constructed as described above except that the 332-bp DNA fragment was replaced by a 145-bp DNA fragment (−345 to −202, containing DRE motifs, Figure 2A) or a 187-bp DNA fragment (−201 to −14, containing MCB motifs, Figure 2A). The resulting plasmids were registered as pKB8606 (Rhp51^DRE^ reporter vector) and pKB8608 (Rhp51^MCB^ reporter vector), respectively. The reporter vector containing three tandem repeats of the MCB motif was constructed as described previously [17], except that the following MCB oligonucleotides were used: sense primer, 5′-GGC TTC GGA CGC GTT ATA CAC GGA CGC GTT ATA CAC ACG GAC GCG TTA GCA C-3′ and antisense primer, 5′- TCG AGT GCA TAA CGC GTC CGT GTG TAT AAC GCG TCC GTG TAT AAC GCG TCC GAA GCC TGC A-3′. The resulting plasmid was registered as pKB8888 (3xMCB reporter vector). The Rhp51^ΔMCB^ reporter vector was constructed using pKB8310 as a template, primer 4730 (5′-CTA GGT AAC AAT TGA TTG AAA TTT AAT TCC TTC ACA ATC CC-3′) as the sense primer, and primer 4731 (5′-GGG ATT GTG AAG GAA TTA AAT TTC AAT CAA TCA ATT GTT ACC TAG-3′) as the antisense primer. The resulting plasmid was registered as pKB8929 (Rhp51^ΔMCB^ reporter vector).

The *cdc18* ^+^ promoter vectors were constructed as described above except that the 663-bp DNA fragment in the 5′ flanking region of the *cdc18* ^+^ gene was amplified using the following PCR primers: sense primer #4684, 5′-AAA ACT GCA GGG GGT TTA TGT TTA GTT TA-3′ and antisense primer #4685, 5′-CCG CTC GAG ATC GAT ACT TTA TAG TAA C-3′. The resulting plasmid was registered as pKB8876 (*cdc18* ^+^ reporter vector).

### Reporter assay in living fission yeast cells

The reporter plasmids were transformed into fission yeast cells as described previously [17]. Cells transformed with the reporter plasmids were cultured at 27°C in Edinburgh Minimal Medium (EMM) to mid-log phase, and the optical density was adjusted to 0.3 at 660 nm. After incubation for 4 h at 27°C, cells (1 mL) were washed twice and resuspended in fresh EMM. Luciferin (L-8240; Biosynth AG) was added to a final concentration of 0.5 mM. Hydroxyurea (HU, 18947-54; Nacalai Tesque) was added to a final concentration of 1 mM, 2 mM, or 4 mM. Emitted light was detected at 1-min intervals using a luminometer (AB-2350; ATTO, Tokyo, Japan) and reported as relative light units (RLUs).

### Fluorescence imaging

Calcofluor White staining was performed as described previously [18]. Microscopic analysis was performed using a microscope (Axioskop 2 Plus; Carl Zeiss, Inc., Germany) equipped with an alpha Plan-Fluar 100x/1.45 oil objective (Carl Zeiss, Inc.). Photographs were taken using a SPOT 2 digital camera in combination with the Spot32 software version 2.1.2 (Diagnostic Instruments, Sterling Heights, MI). The length of the cells in the acquired images was quantified using ImageJ software (http://rsb.info.nih.gov/ij/). Fluorescence images were processed using Corel-DRAW graphics suite version 11.0 (Corel Corporation, Ottawa, Canada) for illustrative purposes only.

### Phos-tag SDS-PAGE and western blotting

To detect the phosphorylation of Cds1, we used fission yeast cells expressing Flag-tagged Cds1 (KP6609 h^-^ *leu1-32 ura4-D18* Cds1-5Flag-kanMX_6_). A PCR-based method [19] was used to generate a *cds1* ^+^::5Flag-kanMX_6_ strain. The yeast cells were treated with HU at various concentrations for 4 h at 27°C, and cell extracts were prepared as previously described [20]. The protein extract was subjected to Phos-tag SDS-PAGE (SuperSep Phos-tag, 50 µM; 10%, Wako) and western blotting using PVDF membranes (Amersham Biosciences). The blotted membrane was incubated with blocking buffer (gelatin) containing an anti-FLAG M2 monoclonal antibody (1∶1000; F1804; Sigma, St. Louis, MO, USA) for 1 h at room temperature. Signals were detected with Clarity Western ECL Substrate (Bio-Rad, Hercules, CA, USA).

### Cell synchronization

A yeast stain carrying the *cdc25-22* allele with a temperature sensitive mutation was used for cell synchronization studies, as described previously [10]. Briefly, *cdc25-22* cells were cultured at a permissive temperature (25°C) in a shaker water bath until mid-log phase, shifted to a non-permissive temperature (36°C) for 4 h, and then cultured at the permissive temperature to release the cell cycle block.

### RNA extraction and quantitative RT-PCR

RNA extraction and quantitative RT-PCR were performed as described previously [21]. Briefly, total RNA was extracted from yeast cells using an RNeasy Mini kit (Qiagen) with on-column deoxyribonuclease digestion (RNase-Free DNase Set; Qiagen). cDNA was synthesized from the resultant total RNA using a High Capacity cDNA Reverse Transcription kit (ABI) and subjected to quantitative PCR with the SYBR Green PCR Master Mix (ABI). The Rhp51 primers for RT-PCR were 5′-TAG TCC GTG TTT GCC TGA GA-3′ (#4984) and 5′-GGG ATC ACC AAC ACC ATC A-3′ (#4985). The Cdc18 primers for RT-PCR were 5′-AGC ATG CTG ATG AAA CAC C-3′ (#4986) and 5′-CTT TCC GGG CAC ATA ATT C-3′ (#4987). Act1 was used as an internal control, and the Act1 primers for RT-PCR were 5′-ATC CAA CCG TGA GAA GAT GA-3′ (#4647) and 5′-ACC ATC ACC AGA GTC CAA GA-3′ (#4648). Signals were detected and analyzed with an Applied Biosystems 7500 Real-Time PCR System (ABI). Data were analyzed according to the comparative C_T_ method.

### Statistical analyses

Data are shown as the mean ± SEM. For comparison of more than two groups, one-way ANOVA was performed followed by Tukey's multiple comparisons test to evaluate pairwise group differences. For comparison of two factors, two-way ANOVA was used. *P*-values less than 0.05 were considered statistically significant. The analyses were performed with PRISM 5.0 software (GraphPad).

## Results and Discussion

### MCB motifs in the *rhp51* ^+^ promoter are responsible for *rhp51* ^+^ transcription upon DNA replication stress

In this study, DNA replication stress was induced by HU, an inhibitor of ribonucleotide reductase that depletes deoxyribonucleotides by suppressing *de novo* nucleotide synthesis [22]–[24]. To check whether the replication checkpoint was activated by the HU concentrations used in this study, we monitored the phosphorylation of Cds1, a checkpoint kinase that is phosphorylated upon DNA replication stress. To this end, Flag-tagged Cds1 was integrated into yeast genome, and the protein was detected with western blot analysis using Phos-tag SDS-PAGE and an anti-Flag antibody. A single Cds1 band was detected in the vehicle condition, but an additional Cds1 band with slower mobility was detected after HU treatment at 2 mM or 4 mM for 4 h (Figure 1A). These signals were not detected in samples from wild-type cells, which did not express Flag-tagged Cds1 (Figure 1A), indicating that the signals were specific to Cds1. These findings showed that DNA replication stress induced by HU treatment stimulated the phosphorylation of Cds1 under the conditions used in this study. We also found that treatment with HU at 2 mM and 4 mM increased the cell length (Figure 1B), a morphological change consistent with cell cycle arrest. These findings suggest that HU treatment activates DNA replication stress, leading to cell cycle arrest.

**Figure 3:**
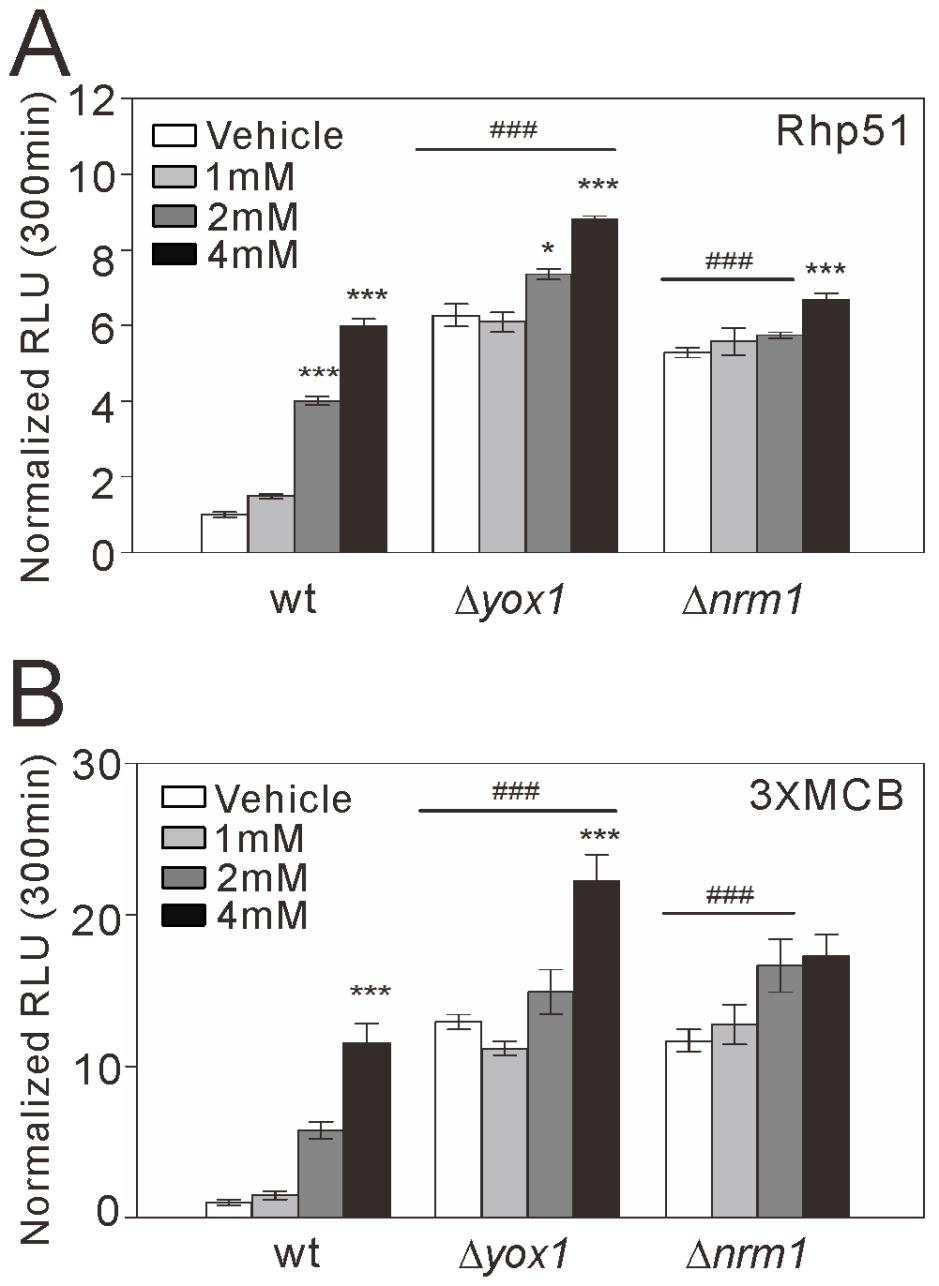
Activities of the full-length *rhp51* ^+^ and 3xMCB reporters in Δ*yox1* and Δ*nrm1* cells treated with HU. Wild-type (wt) cells, Δ*yox1* cells, and Δ*nrm1* cells transformed with the full-length *rhp51* ^+^ (A, “Rhp51”) or 3xMCB (B) reporter were treated with HU at 1 mM, 2 mM, or 4 mM or with vehicle, as described in Figure 2B. The reporter activity at 300 min was analyzed and plotted as described in Figure 2B. *n* = 3 for each group. *P<0.05 and ***P<0.001 compared with the vehicle condition for the respective genotype using one-way ANOVA followed by Tukey's test. ###*P*<0.001 compared with wild-type cells treated with the same HU concentration using one-way ANOVA followed by Tukey's test.

To identify the promoter region involved in the regulation of the *rhp51* ^+^ gene, constructs containing the full-length *rhp51* ^+^ promoter or various *rhp51* ^+^ promoter deletions were generated and subcloned into a luciferase reporter vector (Figure 2A). These plasmids were transformed into wild-type cells, and reporter assays were performed in the presence of the indicated doses of HU. In wild-type cells transformed with the full-length *rhp51* ^+^ reporter vector (pKB8310), treatment with HU increased reporter activity in a dose-dependent manner (Figure 2B, Rhp51). Wild-type cells transformed with the Rhp51^DRE^ reporter vector (pKB8606, the truncated promoter containing DRE1 and DRE2) did not respond to HU treatment (Figure 2B, Rhp51^DRE^). In contrast, the activity of Rhp51^MCB^ reporter vector (pKB8608, the truncated promoter containing MCB motifs) increased in a dose-dependent manner with HU treatment. Its response was similar to that of the full-length promoter, although the basal reporter activity of Rhp51^MCB^ reporter vector was considerably lower (Figure 2B, Rhp51^MCB^). To determine the importance of the MCB motifs, we constructed Rhp51^ΔMCB^ (pKB8929), in which two MCB motifs were removed from the full-length *rhp51* ^+^ reporter. In wild-type cells transformed with the Rhp51^ΔMCB^ reporter vector, HU-induced transcriptional activation was abolished (Figure 2B, Rhp51^ΔMCB^). Furthermore, the activity of a reporter vector containing three tandem repeats of MCB motifs (3xMCB reporter, pKB8888) also increased upon HU treatment (Figure 3B). These results suggest that MCB motifs, but not DREs, are necessary and sufficient for the transcriptional induction of the *rhp51* ^+^ gene in response to DNA replication stress.

### MBF co-repressors, Yox1 and Nrm1, are involved in DNA replication stress-induced activation of the *rhp51* ^+^ promoter

Because the MBF co-repressors, Nrm1 and Yox1, regulate MBF-dependent transcription in fission yeast [8], [9], [25], [26], we examined their roles in *rhp51* ^+^ transcription induced by DNA replication stress. In the vehicle condition, the activity of the full-length *rhp51* ^+^ reporter vector was higher in Δ*yox1* and Δ*nrm1* cells than in wild-type cells (Figure 3A), suggesting that Yox1 and Nrm1 constitutively repress the *rhp51* ^+^ promoter. The increase mostly precluded HU-induced *rhp51* ^+^ transcription in both Δ*yox1* and Δ*nrm1* cells, thus implicating these MBF co-repressors in this process. Given the role of MCB motifs in HU-induced *rhp51* ^+^ transcription, we also examined the activity of the 3xMCB reporter, described above, in Δ*yox1* and Δ*nrm1* cells without or with HU treatment (Figure 3B). Similar to the activity of the full-length *rhp51* ^+^ reporter, 3xMCB reporter activity increased in the absence of HU treatment when Yox1 and Nrm1 were deleted, and the increase mostly precluded the HU-induced activation of the reporter. These results demonstrate a role for the MBF co-repressors Yox1 and Nrm1 in regulating *rhp51* ^+^ transcription in the absence or presence of DNA replication stress. Furthermore, these results suggest that DNA replication stress releases MBF-regulated *rhp51* ^+^ transcription from Yox1/Nrm1-mediated repression.

### The Rad3-Cds1/Chk1 pathway partially mediates *rhp51^+^* transcription upon DNA replication stress

**Figure 4:**
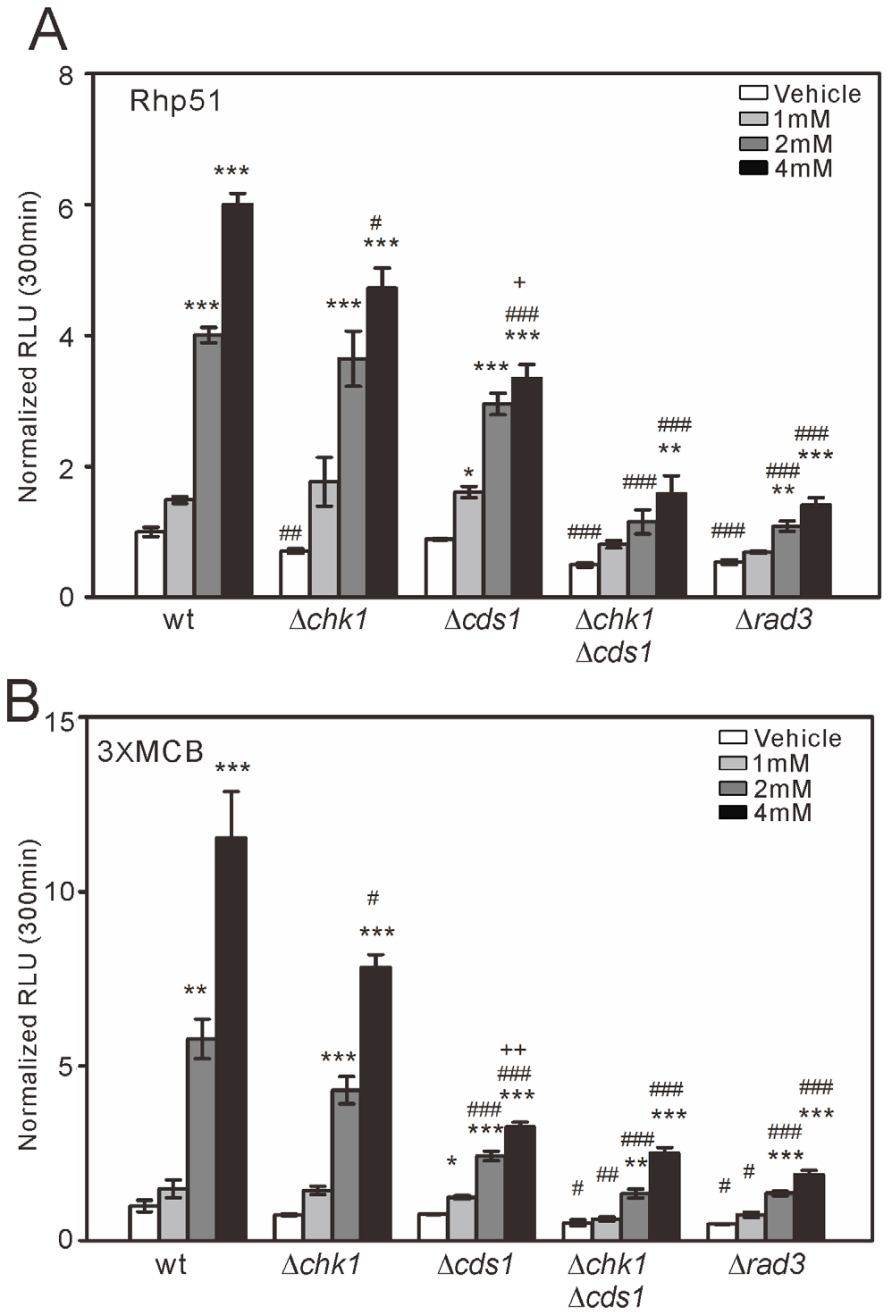
Activities of the full-length *rhp51* ^+^ and 3xMCB reporters in Δ*chk1*, Δ*cds1*, Δ*chk1*Δ*cds1*, and Δ*rad3* cells treated with HU. Cells transformed with the full-length *rhp51* ^+^ (A, “Rhp51”) or 3xMCB (B) reporter were treated with HU at 1 mM, 2 mM, or 4 mM or with vehicle, as described in Figure 2B. The reporter activity at 300 min was analyzed and plotted as described in Figure 2B. *n* = 3 for each group. *P<0.05, **P<0.01, and ***P<0.001 compared with the vehicle condition for the respective genotype using one-way ANOVA. #*P*<0.05, ##*P*<0.01, and ###*P*<0.001 compared with wild-type cells treated with the same HU concentration using one-way ANOVA. +*P*<0.05 and ++*P*<0.01 compared with Δ*chk1* cells treated with the same HU concentration using one-way ANOVA.

Because the Rad3-Cds1/Chk1 pathway inhibits Nrm1, thereby de-repressing G1/S transcription upon DNA replication stress [8]–[12], we examined whether the pathway was involved in HU-induced *rhp51* ^+^ transcription using cells deficient in this pathway. In Δ*cds1* and Δ*chk1* cells, *rhp51* ^+^ transcription was modestly reduced by treatment with 4 mM HU (Figure 4A), but to a statistically significant extent. The levels of *rhp51* ^+^ transcription in Δ*cds1* cells were significantly lower than those in Δ*chk1* cells (Figure 4A). HU-induced *rhp51* ^+^ transcription was reduced to a greater extent in Δ*chk1*Δ*cds1* cells than in either of the single knockout cells (Figure 4A), suggesting that the two kinases have compensatory roles. In addition, HU-induced *rhp51* ^+^ transcription was reduced in Δ*rad3* cells to a level comparable to that in Δ*chk1*Δ*cds1* cells (Figure 4A). However, HU treatment still induced *rhp51* ^+^ transcription in both Δ*chk1*Δ*cds1* cells and Δ*rad3* cells, albeit to a lesser extent than in wild-type cells. We also examined the activity of the 3xMCB reporter in these cells. The results were similar to those obtained using the *rhp51* ^+^ reporter. Thus, in Δ*chk1*Δ*cds1* cells and Δ*rad3* cells, HU-induced reporter activation was attenuated, but not abolished (Figure 4B). These results showed that the Rad3-Cds1/Chk1 pathway plays a critical role in the transcription of *rhp51* ^+^ mediated by MCB motifs upon DNA replication stress, but they also suggest that an unidentified checkpoint signaling pathway, in addition to the Rad3-Cds1/Chk1 pathway, regulates the *rhp51* ^+^ transcription.

### DNA replication stress maintains *rhp51* ^+^ transcription beyond the G1/S transition

**Figure 5:**
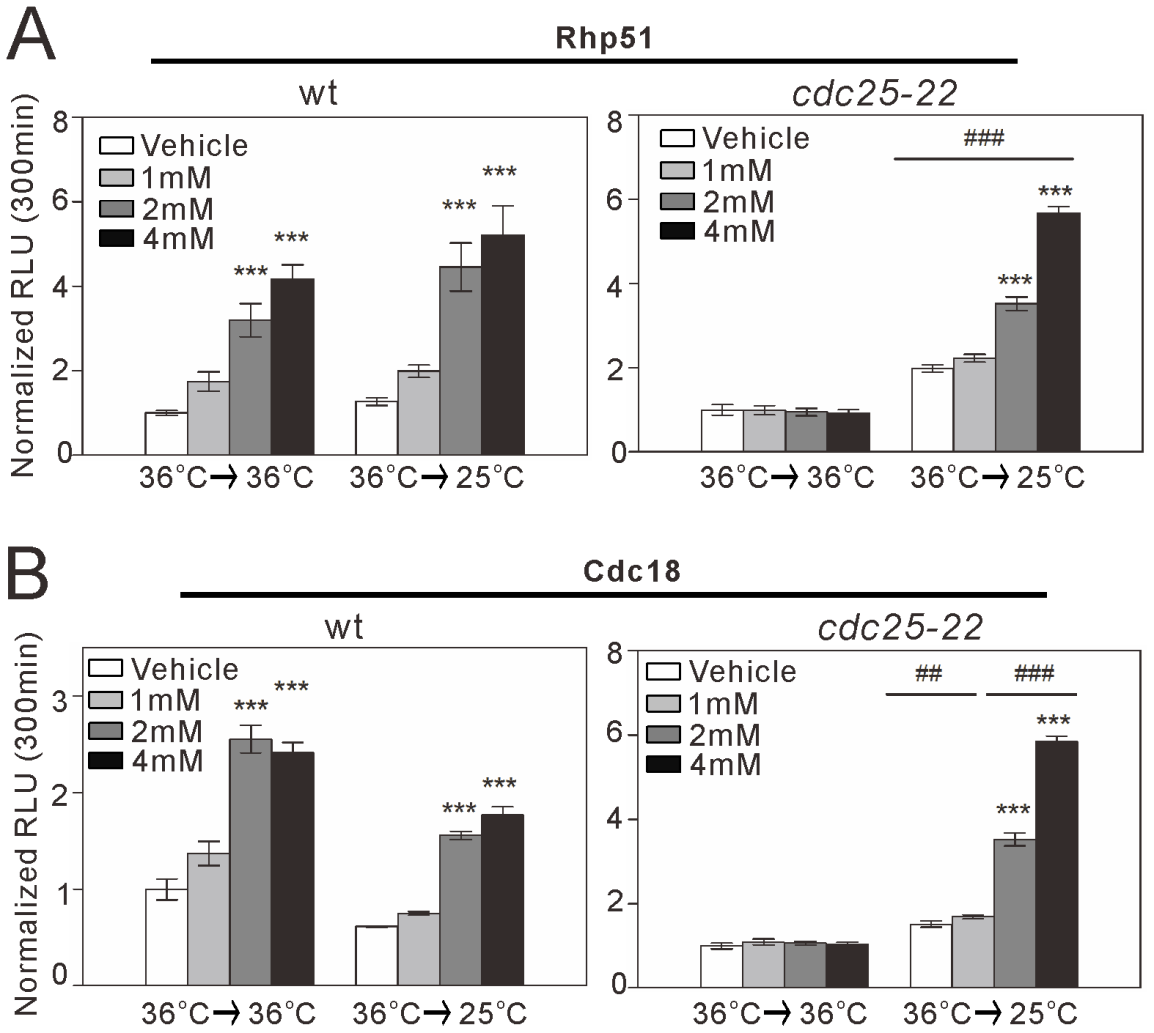
*rhp51* ^+^ and *cdc18* ^+^ transcription in synchronized *cdc25-22* cells treated with HU. Wild-type cells transformed with the full-length *rhp51* ^+^ (A) or *cdc18* ^+^ (B) reporter were cultured to mid-log phase at 25°C in EMM and shifted to 36°C for 4 h. The cells were then maintained at 36°C continuously for G2 block (“36°C→36°C”) or shifted to 25°C for block and release (“36°C→25°C”). The cells were treated with HU at 1 mM, 2 mM, or 4 mM or with vehicle, as described in Figure 2B. The reporter activity at 300 min was analyzed and plotted as described in Figure 2B. *n* = 8 and 4 for each group in the block condition and the block and release condition, respectively. ***P<0.001 compared with the vehicle condition for the respective genotype and temperature condition using one-way ANOVA. ##*P*<0.01 and ###*P*<0.001 compared with G2 block at the same HU concentration using one-way ANOVA.

Because MBF and its co-repressors are critical for G1/S transcription, we examined the cell cycle regulation of *rhp51* ^+^ transcription and its modulation by DNA replication stress. To this end, we used *cdc25-22* cells, which arrested at G2 phase at a restrictive temperature (36°C) and progressed through the cell cycle when shifted to a permissive temperature (25°C). We examined *rhp51* ^+^ reporter activity in *cdc25-22* cells during G2 block (continuous 36°C culture) or after block and release (36°C culture for 4 h followed by a shift to 25°C). We also examined the activity of the *cdc18* ^+^ promoter using a luciferase reporter vector because *cdc18* ^+^ is regulated by both G1/S transcription and DNA replication stress. The *rhp51* ^+^ reporter showed HU-induced activation in wild-type cells under nominal G2 block and block and release conditions (Figure 5A, left panel). In contrast, in *cdc25-22* cells, the HU-induced increase in *rhp51* ^+^ transcription was abolished under G2 block conditions, but was observed under block and release conditions (Figure 5A, right panel). We also examined the activity of Rhp51^MCB^ and Rhp51^DRE^ in synchronized *cdc25-22* cells. Rhp51^MCB^, but not Rhp51^DRE^, showed HU-induced increases in reporter activity, similar to the full-length promoter (Figure S1). The cycle dependency of HU-induced *rhp51* ^+^ and *cdc18* ^+^ transcription is reasonable because HU-induced DNA replication stress should occur, in principle, only during S phase. Similar to the activity of the full-length *rhp51* ^+^ promoter, *cdc18* ^+^ reporter activity increased in response to HU treatment under block and release conditions, but not under G2 block conditions (Figure 5B).

**Figure 6:**
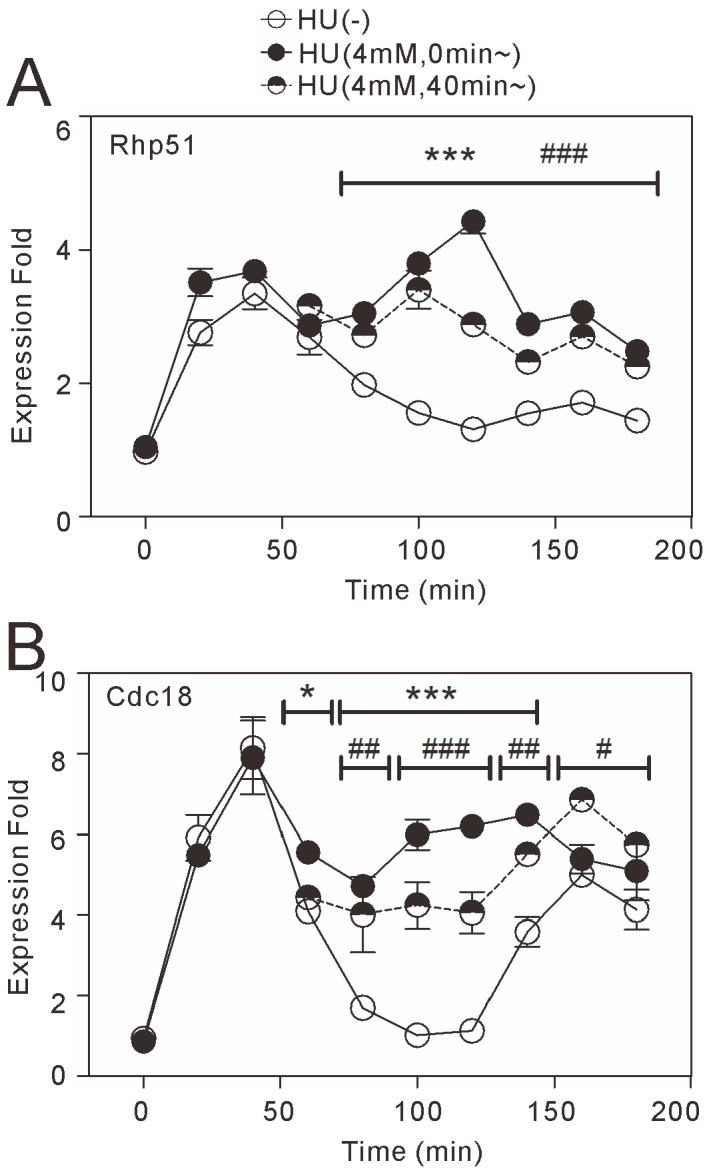
Analysis of Rhp51 and Cdc18 mRNA levels in synchronized *cdc25-22* cells treated with HU. The *cdc25-22* cells were synchronized as described in Figure 5. The cells were divided into three groups of equal volume, of which one was left untreated (white circles) and two were incubated with 4 mM HU from 0 min (black circles) or from 40 min (black/white circles) after the release from G2 block. Total RNA was extracted from aliquots of equal volume collected from the culture every 20 min and subjected to quantitative RT-PCR analysis. The traces in the graphs show the mRNA levels of Rhp51 and Cdc18 averaged across three independent samples at each respective time point. *P<0.05 and ***P<0.001 for comparison of HU treatment from 0 min with vehicle treatment. #*P*<0.05, ##*P*<0.01, and ###*P*<0.001 for comparison of HU treatment from 40 min with vehicle treatment. Statistical analyses were performed using two-way ANOVA followed by Bonferroni's multiple comparisons test.

To test whether our findings with the luciferase reporter assay extended to the regulation of endogenous promoters, we used qRT-PCR to examine the mRNA levels of *rhp51* ^+^ and *cdc18* ^+^ in synchronized *cdc25-22* cells with or without HU treatment. Without HU treatment, the mRNA levels of *rhp51* ^+^ and *cdc18* ^+^ increased at 20–60 min and returned towards baseline at 100–140 min after the release from G2 block (Figure 6). This result indicates that *rhp51* ^+^ transcription, similar to *cdc18* ^+^ transcription, is induced at the G1/S transition. With 4 mM HU treatment, the initial peak in *rhp51* ^+^ transcription at 20–60 min was not affected, but the increased level was maintained during the period when *rhp51* ^+^ transcription returned to baseline without HU treatment (80–180 min) (Figure 6). To examine the cause of the delayed HU action, HU was applied at 40 min, the time when *rhp51* ^+^ transcription peaked without HU treatment. Interestingly, HU treatment at this time point maintained *rhp51* ^+^ transcription from 80 min after the release from G2 block, as early as when HU treatment was applied at the beginning of the experiment. This finding indicates that DNA replication stress maintains the G1/S transcription of *rhp51* ^+^ beyond the entry into S phase. As observed with *rhp51* ^+^ transcription, the initial peak in *cdc18* ^+^ transcription at the G1/S transition was maintained throughout the observation period in cells treated with HU (Figure 6). These results showed that *rhp51* ^+^ transcription associated with the G1/S transition was maintained by DNA replication stress, similar to MBF-dependent *cdc18* ^+^ transcription.

Taken together, our results showed that MCB motifs, but not DREs, in the *rhp51* ^+^ promoter mediated *rhp51* ^+^ transcription upon DNA replication stress. Consistent with this finding, *rhp51* ^+^ transcription was suppressed by the MBF co-repressors Yox1 and Nrm1, and DNA replication stress de-repressed and maintained MBF-mediated *rhp51* ^+^ transcription beyond the G1/S transition. The transcription of *rhp51* ^+^ was similar to that of *cdc18* ^+^, which was induced at the G1/S transition and maintained by DNA replication stress, as previously reported [8] and confirmed in the present study. Therefore, our findings support the idea that DNA replication stress de-represses MBF-dependent G1/S transcription at most, if not all, target genes. DNA replication stress induced *rhp51* ^+^ transcription primarily through the Rad3-Cds1/Chk1 pathway. However, given that DNA replication stress was able to induce *rhp51* ^+^ transcription in the absence of the Rad3-Cds1/Chk1 pathway, another checkpoint pathway may regulate *rhp51* ^+^ transcription upon DNA replication stress. Although it has been reported that DNA damage induces *rhp51* ^+^ transcription through the DREs in its promoter [14], this study showed that DNA replication stress induced *rhp51* ^+^ transcription in the absence of DREs. However, because the truncated *rhp51* ^+^ promoter without DREs (Rhp51^MCB^) showed significantly lower activity with or without HU treatment when compared with the activity of the full-length promoter, a role for DREs in maintaining *rhp51* ^+^ transcription has not been excluded. It is plausible that DNA damage and DNA replication stress activate distinct transcription factors to induce *rhp51* ^+^ transcription. Thus, whether similar MBF-dependent mechanisms operate and interact with DRE-bound transcription factors to induce *rhp51* ^+^ transcription upon DNA damage warrants future investigation.

## Supporting Information

Figure S1 **Reporter analysis with full-length and truncated *rhp51*^+^ promoters in wild-type and synchronized *cdc25-2*2 cells treated with HU.** Wild-type and *cdc25-22* cells transformed with the full-length *rhp51* ^+^, Rhp51^MCB^, or Rhp51^DRE^ reporter were cultured as described in Figure 5. The cells were treated with HU at 1 mM, 2 mM, or 4 mM or with vehicle, as described in Figure 2B. Reporter activity was analyzed and plotted as described in Figure 2B. *n* = 4 for each group. **P<0.01 and ***P<0.001 compared with vehicle treatment using one-way ANOVA followed by Tukey's test.
(TIF)
