# Supplementary figures and images for: The MluI Cell Cycle Box (MCB) Motifs, but Not Damage-Responsive Elements (DREs), Are Responsible for the Transcriptional Induction of the rhp51 + Gene in Response to DNA Replication Stress
Source: PLoS One. 2014 Nov 5;9(11):e111936. doi: 10.1371/journal.pone.0111936 (PMC4221157; doi:10.1371/journal.pone.0111936)

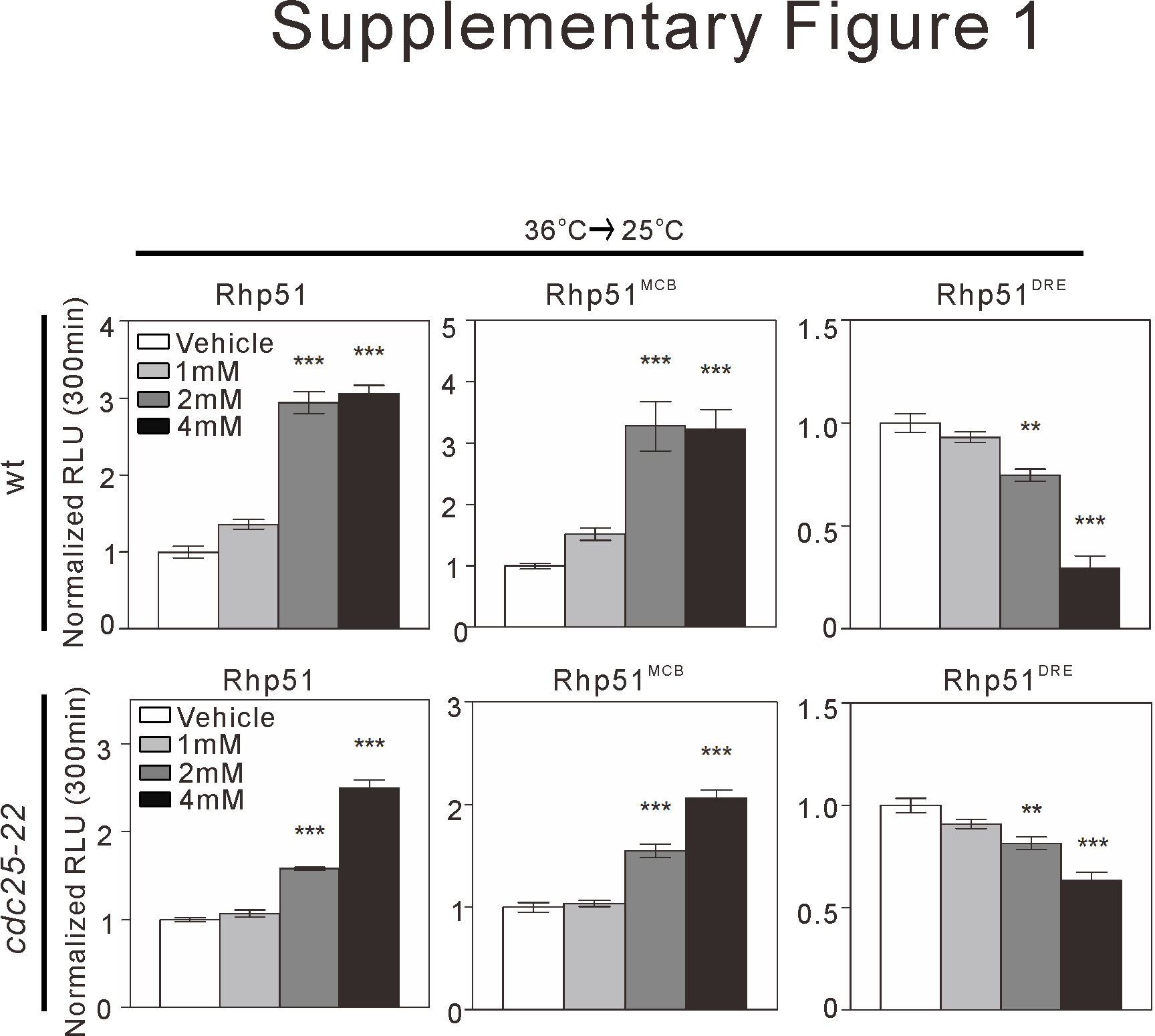

Supplement: Figure S1 — Reporter analysis with full-length and truncated rhp51+ promoters in wild-type and synchronized cdc25-22 cells treated with HU. Wild-type and cdc25-22 cells transformed with the full-length rhp51 +, Rhp51MCB, or Rhp51DRE reporter were cultured as described in Figure 5. The cells were treated with HU at 1 mM, 2 mM, or 4 mM or with vehicle, as described in Figure 2B. Reporter activity was analyzed and plotted as described in Figure 2B. n = 4 for each group. **P<0.01 and ***P<0.001 compared with vehicle treatment using one-way ANOVA followed by Tukey's test. (TIF) [file pone.0111936.s001.tif]
